# Feed Restriction Alleviates Chronic Thermal Stress-Induced Liver Oxidation and Damages via Reducing Lipid Accumulation in Channel Catfish (*Ictalurus punctatus*)

**DOI:** 10.3390/antiox11050980

**Published:** 2022-05-17

**Authors:** Qisheng Lu, Yulong Gong, Longwei Xi, Yulong Liu, Wenjie Xu, Haokun Liu, Junyan Jin, Zhimin Zhang, Yunxia Yang, Xiaoming Zhu, Shouqi Xie, Dong Han

**Affiliations:** 1 State Key Laboratory of Freshwater Ecology and Biotechnology, Institute of Hydrobiology, Chinese Academy of Sciences, Wuhan 430072, China; luqisheng@ihb.ac.cn (Q.L.); gongyl@ihb.ac.cn (Y.G.); xilw@ihb.ac.cn (L.X.); liuyulong@ihb.ac.cn (Y.L.); xuwenjie@gdou.edu.cn (W.X.); liuhaokun@ihb.ac.cn (H.L.); jinjunyan@ihb.ac.cn (J.J.); zhangzm@ihb.ac.cn (Z.Z.); yxyang@ihb.ac.cn (Y.Y.); xmzhu@ihb.ac.cn (X.Z.); sqxie@ihb.ac.cn (S.X.); 2 College of Advanced Agricultural Sciences, University of Chinese Academy of Sciences, Beijing 100049, China; 3 The Innovative Academy of Seed Design, Chinese Academy of Sciences, Wuhan 430072, China; 4 Hubei Engineering Research Center for Aquatic Animal Nutrition and Feed, Wuhan 430072, China

**Keywords:** thermal stress, oxidative damage, liver health, feed restriction, channel catfish

## Abstract

Caloric restriction is known to suppress oxidative stress in organ systems. However, whether caloric/feed restriction alleviates chronic thermal stress in aquatic animals remains unknown. Here, we set up three feeding rations: 3% BW (3% body weight/day), 2.5% BW (restricted feeding, 2.5% body weight/day) and 2% BW (high restricted feeding, 2% body weight/day), to investigate the effects and mechanism of feed restriction on improving chronic heat-induced (27 to 31 °C) liver peroxidation and damages in channel catfish (*Ictalurus punctatus*). The results showed that, compared to 3% BW, both 2.5% BW and 2% BW significantly reduced the liver expressions of hsc70, hsp70 and hsp90, but only 2.5% BW did not reduce the growth performance of channel catfish. The 2.5% BW and 2% BW also reduced the lipid deposition (TG) and improved the antioxidant capacity (CAT, SOD, GSH and T-AOC) in the liver of channel catfish. The heat-induced stress response (plasma glucose, cortisol and NO) and peroxidation (ROS and MDA) were also suppressed by either 2.5% BW or 2% BW. Moreover, 2.5% BW or 2% BW overtly alleviated liver inflammation and damages by reducing endoplasmic reticulum (ER) stress (BIP and Calnexin) and cell apoptosis (BAX, Caspase 3 and Caspase 9) in the liver of channel catfish. In conclusion, 2.5% body weight/day is recommended to improve the antioxidant capacity and liver health of channel catfish during the summer season, as it alleviates liver peroxidation and damages via suppressing lipid accumulation under chronic thermal stress.

## 1. Introduction

Temperature is a crucial environmental factor that strictly affects the metabolism, immunology and stress state of fish [1,2]. Aquatic animals suffer temperature fluctuations in their aquatic environments. As ectotherms, the body temperature of fish is closely influenced by the temperature of the surrounding water [3]. In summer, aquaculture species usually experience a high-temperature period for 2–3 months. The suitably higher water temperatures are beneficial for maximizing growth performance and improving the immune systems in aquaculture species [4]. However, the persistent extremely high temperature deteriorates due to a chronic thermal stress, which usually leads to tissue oxidative damages and metabolism disorders in cultured fish [5,6]. When the temperature rises above the threshold for one species, it may cause physiological disturbances and even lead to fish death [7]. High temperature-induced thermal stress in cultured fish has been an obvious threat for the development of aquaculture. Therefore, exploring the available strategies is imperative for mitigating the negative effects of thermal stress on cultured fish.

As a regular approach, food restriction has been investigated to improve health spans in different organisms by enhancing their antioxidant and immunology capacities [8,9]. A previous study showed that food restriction increased the survival of rodents by altering the activities of antioxidant enzymes and reducing free radical damages in various tissues [10]. Importantly, food restriction reduced the food frequency-induced increase in mitochondrial oxidative stress and apoptosis in the central nervous system [11]. A recent study in humans also demonstrated that sustained food restriction activated a core transcriptional program that promoted the immune system and reduced inflammation [8]. Moreover, food restriction imitated vitamin E in the promotion of insulin secretion and glycemic homeostasis, which was speculated to be involved in antioxidant effects [12]. These studies indicate that food restriction is an effective experimental intervention for enhancing antioxidant capacity and attenuating oxidative stress. Consequently, appropriate feed restriction may be one of the potential solutions for alleviating high temperature-induced thermal stress and peroxidation in cultured aquatic animals.

Stressors such as high-temperature, excessive food intake and high stocking density can cause oxidative stress induced by the accumulation of reactive oxygen species (ROS) [13,14,15], which leads to lipid peroxidation, protein carboxylation and nucleic acid damage [16]. As a consequence, oxidative stress negatively regulates the growth performance [17,18] and the immune system in fish [19]. Therefore, investigations for improving the antioxidation capacity and health state of aquaculture fish have become more and more popular. In recent years, efforts to reduce peroxidation and stress by dietary adjustment [20], feed additives [21,22] and feeding strategies [23] have successfully improved the antioxidation systems of fish. Feed restriction was one of the feeding strategies that contributed to the skeletal muscle growth [24] and lipid consumption [25] in fish. Interestingly, feed restriction also mitigated high carbohydrate-induced oxidative stress and inflammation via motivating the AMPK-SIRT1 pathway [26]. That study determined the possibility of improving antioxidation and anti-stress capacities through feed restriction. However, whether feed restriction alleviates the chronic high temperature-induced oxidative stress and its underlying regulation mechanisms remains unanswered.

Channel catfish (*Ictalurus punctatus*) originates from North America and has become one of the most popular cultured catfish species. The production of channel catfish exceeded 390,000 tons with an increase of 3.34% over recent years worldwide [27]. However, thermal stress and oxidative stress have been major challenges for the health and survival of cultured channel catfish during the summer season. Therefore, we set out to investigate the effects and mechanism of feed restriction on the alleviation of high temperature-induced oxidative stress and liver damages of channel catfish. Using three feeding rations (3% BW, 2.5% BW and 2% BW), we confirmed the positive effects and regulation mechanism of appropriate feed restriction on the antioxidant capacity of cultured channel catfish under thermal stress. These findings provide a feasible solution for alleviating high temperature-induced thermal stress and peroxidation in cultured aquatic animals.

## 2. Materials and Methods

### 2.1. Ethics Statement

Procedures related to animal treatments in this study were conducted strictly according to the Guiding Principles for the Care and Use of Laboratory Animals and were approved by the Institute of Hydrobiology, Chinese Academy of Sciences (Approval ID: IHB 2013724).

### 2.2. Channel Catfish and Feeding Trial

Juvenile channel catfish were provided by Wuhan Dabeinong Technology Co., Ltd. (Wuhan, China). All fish were cultured in an experimental system with commercial feed for 2 weeks to adapt to the experimental conditions. Before the feeding trial, fish were fasted for 24 h for gastric emptying. Then, 80 channel catfish (initial weight: 35.5 ± 0.1 g) were randomly selected, weighed and assigned into each floating net cage. Six replicates were randomly distributed for each group. During the experiment, fish were fed twice a day (08:30 and 16:30). Commercial feed (crude protein, 35.5%; crude lipid, 9.0%; moisture, 9.6%; ash, 10.4%) (Wuhan Dabeinong Technology Co., Ltd., Wuhan, China) was used for feeding the fish. The daily feeding rations of each floating net cage were 3% BW (3% body weight/day), 2.5% BW (restricted feeding, 2.5% body weight/day) and 2% BW (high restricted feeding, 2% body weight/day), respectively. The 3% BW is considered an approximate satiation according to the previous studies [22,28]. The feeding trial lasted for 13 weeks. The whole experiment was carried out in a natural environment. Throughout the whole trial, the water temperature was detected by a thermometer everyday, the dissolved oxygen content of the water was maintained at 6.4 ± 0.5 mg/L, and the ammonia-N content of the water was lower than 0.5 mg/L.

### 2.3. Sample Collection

After a 24-h fasting, growth performance was calculated, and the fish were anesthetized with MS-222 (80 mg/L, Sigma Aldrich Co. LLC., St. Louis, MO, USA) before sampling [29]. Three fish were randomly taken from each floating net cage for tail vein blood collection, which was collected in a 1.5 mL centrifuge tube moistened with 0.2% sodium heparin and centrifuged at 3500 rpm for 10 min. Then, the supernatant was collected and stored at −80 °C. Another three fish from each floating net cage were quickly dissected on ice, and liver tissue was removed, placed onto enzyme-free tinfoil and put in liquid nitrogen before storing at −80 °C.

### 2.4. Determination of Growth Parameters

The weight gain rate (WG), specific growth rate (SGR), feed efficiency (FE), feeding rate (FR), survival rate (SR), hepatosomatic index (HSI), viscerosomatic index (VSI) and condition factor (CF) were calculated as follows: 

WG (%) = (final body weight − initial body weight)/initial body weight × 100

SGR (%/d) = [Ln (final body weight) − Ln (initial body weight)]/days × 100

FE (%) = (final body weight − initial body weight)/dry feed intake × 100

FR (%BW/d) = dry feed intake/[days × (initial body weight + final body weight)/2] × 100

SR (%) = final number of fish/initial number fish × 100

HSI (%) = liver weight/whole body weight × 100

VSI (%) = visceral weight/whole body weight × 100

CF (g/cm^3^) = whole body weight/(body length)^3^ × 100

### 2.5. Biochemical and Antioxidative Parameters

Glucose, alanine aminotransferase (ALT), malondialdehyde (MDA) and the cortisol content (ELISA) in plasma were assayed by commercial assay kits (Nanjing Jiancheng Bioengineering Institute, Nanjing, China, Catalog: A154-1-1, C009-2-1, A003-1-2 and H094). The measurement of liver enzyme activity needed experimental pretreatment. Physiological saline was added to the liver samples as weight (g): volume (mL) = 1:9. After centrifugation, the supernatant was taken out for subsequent enzyme activity analysis. Protein concentrations in the liver homogenates were determined by the Coomassie brilliant blue method (Beyotime, Shanghai, China, P0006). The activities of catalase (CAT), reduced glutathione (GSH) and superoxide dismutase (SOD), as well as the total antioxidant capacity (T-AOC), the content of malondialdehyde (MDA), the nitric oxide (NO) content and the lipid peroxide (LPO) content in the liver samples were determined by enzymatic colorimetric methods (Nanjing Jiancheng Bioengineering Institute, Nanjing, China, Catalog: A007-1-1, A006-2-1, A001-3-2, A015-2-1, A003-1-2, A013-2-1 and A106-1-2). Plasma and liver ROS contents were determined by a fish ELISA kit (Wuhan MSK Biological Technology Co., Ltd., Wuhan, China, 69-86537).

### 2.6. Detection of Lipid Metabolism-Related Enzyme Activity in Liver

The content of liver acetyl-CoA carboxylase (ACC), lipoprotein lipase (LPL) and carnitine palmitoyltransferase 1A (CPT1A) were detected by enzyme-linked ELISA, provided by MSK Biological Technology Co., Ltd. (Wuhan, China, Catalog: 69-47369, 69-20008 and 69-22482). Triglycerides (TG) and lipase (LPS) in the liver samples were measured through colorimetric methods according to the manufacturer’s instructions (Nanjing Jiancheng Bioengineering Institute, Nanjing, China, Catalog: A110-1-1 and A054-1-1).

### 2.7. Apoptosis Detection

The activities of Caspase 3 and Caspase 9 in the liver samples were determined following the protocol for apoptosis detection kits (Beyotime, Shanghai, China, Catalog: C1116 and C1158). The protein concentration in the sample to be tested was detected by the Bradford method (Beyotime, Shanghai, China, P0006), and the enzyme activity units of Caspase 3 and Caspase 9 contained in a unit weight protein of the sample were calculated.

### 2.8. Tissue Total RNA Extraction and RT-qPCR Analysis

Total RNA from the liver tissue was isolated with TRIzol Reagent (Ambion Life Technologies, Carlsbad, CA, USA), following the product manual, and the quality was tested according to the method of Sun et al. [30]. Reverse transcription was performed using M-MLV First-Strand Synthesis Kit (Invitrogen, Shanghai, China), and the cDNA was stored at −20 °C for real-time quantitative PCR (RT-qPCR) analysis.

The real-time quantitative PCR was performed on the LightCycle^®^ 480 II system to determine the amplification efficiency in a pre-experiment and to make standard curves using internal reference and target genes. Light Cycle 480 SYBR Green I Master Mix (Roche, Basel, Switzerland) was used to determine the expression levels of target genes. The fluorescent qPCR reaction solution included 3 µL of LightCycle^®^ 480 SYBR^®^ Green I Master, 0.24 µL of PCR forward primer (10 µM), 0.24 µL of PCR reverse primer (10 µM), 2.0 µL of RT reaction (cDNA solution) and 0.52 µL of dH_2_O. In this study, there was no difference in the expression of β-actin among the three treatments. After analysis, it was chosen as an internal reference for normalization. The qPCR primers were designed using the National Center for Biotechnology Information (NCBI) primer BLAST service. The primers used for RT-qPCR are shown in Table 1. The thermal profile was as follows: 95 °C for 5 min, followed by 45 cycles at 95 °C for 10 s, 60 °C for 20 s and 72 °C for 10 s. Each sample was run in duplicate, and the results were calculated with the means. The expression of each target gene for the different treatment groups was expressed relative to the 3% BW group, and the expression levels of the target genes were calculated by the 2^−∆∆CT^ method [31].

### 2.9. Protein Extraction and Western Blot Analysis

The liver samples were cell lysed by RIPA lysis buffer (Beyotime, Shanghai, China, P0013B) containing a protease inhibitor cocktail and phosphatase inhibitor cocktail (Roche, Basel, Switzerland). The lysates were sonicated and homogenized on ice, centrifuged at 13,000× *g* at 4 °C for 20 min, and the supernatant was collected for later analysis. The sample protein concentrations were determined according to the instructions of the BCA Protein Quantification Kit (Beyotime, Shanghai, China, P0012) and adjusted to a consistent protein concentration for all liver samples using 50% RIPA lysis buffer. After determination of the protein quantity, an equal volume of 5 × sample loading buffer was added and boiled at 100 °C for 5 min. Proteins (20 mg) were loaded into the wells of sodium dodecyl sulfatepolyacrylamide gel electrophoresis (SDS-PAGE gels) and then transferred to polyvinylidene fluoride (PVDF) membranes. The membranes were blocked for 1 h at room temperature by using 5% skimmed milk in TBST buffer (20 mM Tris-HCl, 150 mM sodium chloride, 0.1% Tween 20, pH 7.5) and were then incubated at 4 °C for 12 h using the following specific primary antibodies: Calnexin Antibody (ET1611-86, HUABIO, Hangzhou, China), BIP Antibody (3183S, Cell signaling, Danvers, MA, USA), BAX Antibody (ET1603-34, HUABIO, Hangzhou, China) or β-Actin (4970S, Cell signaling, Danvers, MA, USA). After washing, membranes were incubated with a secondary antibody, Anti-rabbit lgG (7074P2, Cell signaling, Danvers, MA, USA), at room temperature for 2 h. Wherein, β-Actin was used as an internal reference protein. The bands were imaged by ImageQuant LAS 4000mini (GE Healthcare Life Sciences) and quantified using Image J software (National Institutes of Health, Bethesda, MD, USA) [32].

### 2.10. Hematoxylin and Eosin (H&E) Staining

There were six replicates in each treatment group, and samples of the liver tissues were isolated and kept in 4% paraformaldehyde for 24 h. After that, the samples were dehydrated in gradient alcohol dehydration, impregnated with methyl salicylate, embedded in paraffin and cut into 5 μm by a Leica RM 2135 slicing machine (Leica Company, Wetzlar, Germany) followed by H&E staining [33]. The morphological structure of the liver tissues was observed and imaged with a Zeiss microscope (Axioplan-2 imaging, Oberkochen, Germany). The number of macrophages was counted manually.

### 2.11. Statistical Analysis

All data were compared by a one-way analysis of variance (ANOVA) and differences between the groups were tested by Duncan’s multiple-range test. All results are expressed as the “mean ± SEM (standard error of the mean)” of four or six replicates, and all statistical analyses were performed using SPSS 22.0 (IBM, Armonk, NY, USA). Differences were considered significant at *p* < 0.05.

## 3. Results

### 3.1. Feed Restriction (2.5% BW) Suppressed the Thermal Stress Response of Liver without Causing Growth Retardation

**Figure 1:**
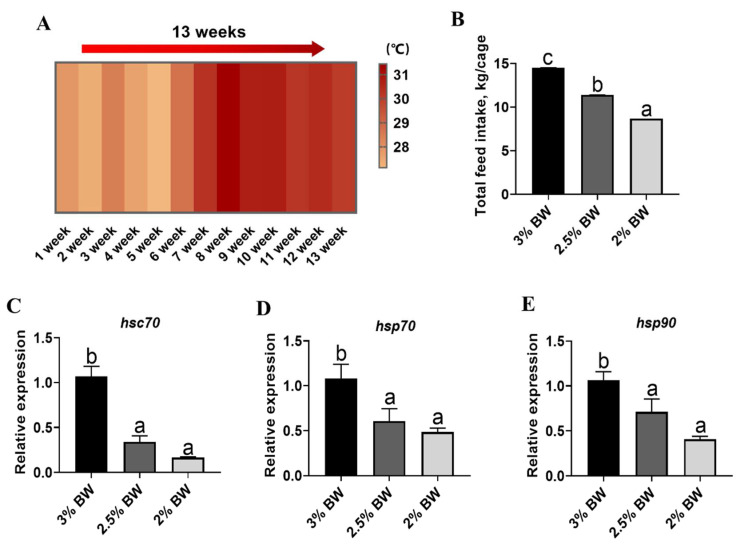
Feed restriction suppressed the heat stress response in livers of channel catfish. (**A**) Water temperature during feeding trial; (**B**) total feed intake during feeding trial; (**C**) liver transcription level of *hsc70*; (**D**) liver transcription level of *hsp70*; (**E**) liver transcription level of *hsp90*. Each data point represents the means ± SEM of six replicates. Significance was evaluated by a one-way ANOVA (*p* < 0.05), followed by Duncan’s multiple range tests. Values marked with different letters (a, b and c) are significantly different between the treatment groups.

During the entire feeding trial, the water temperature gradually increased from 27 °C to 31 °C in the first 8 weeks, and then it was stable at around 30 °C for the remaining 5 weeks (Figure 1A). The total feed intake and the feeding rate significantly decreased among the 3% BW, 2.5% BW and 2% BW groups (*p* < 0.05) (Figure 1B; Table 2). Compared to those of the 3% BW group, the final body weight, weight gain rate (WG) and specific growth rate (SGR) all significantly decreased in the 2% BW group (*p* < 0.05), while there was no obvious decrease in those of the 2.5% BW group (*p* > 0.05) (2). Moreover, the feeding efficiencies (FE) significantly increased both in the 2.5% BW and 2% BW groups (*p* < 0.05) (Table 2). Moreover, the hepatosomatic index (HSI) and condition factor (CF) both significantly decreased in the 2.5% BW group (*p* < 0.05), while the HSI and viscerosomatic index (VSI) both significantly decreased in the 2% BW group (*p* < 0.05) (Table 3). Importantly, we found that the transcription levels of the liver heat shock proteins, heat shock cognate 70 (*hsc70*), heat shock protein 70 (*hsp70*) and heat shock protein 90 (*hsp90*), were all significantly down-regulated by either the 2.5% BW or 2% BW feeding ration (*p* < 0.05) (Figure 1C–E). These data indicate that the appropriate feed restriction (2.5% BW) suppresses the thermal stress response of liver tissues without causing negative effects on the growth performance in channel catfish under a high water temperature.

### 3.2. Feed Restriction Reduced Liver Lipid Accumulation and β-Oxidation

**Figure 2:**
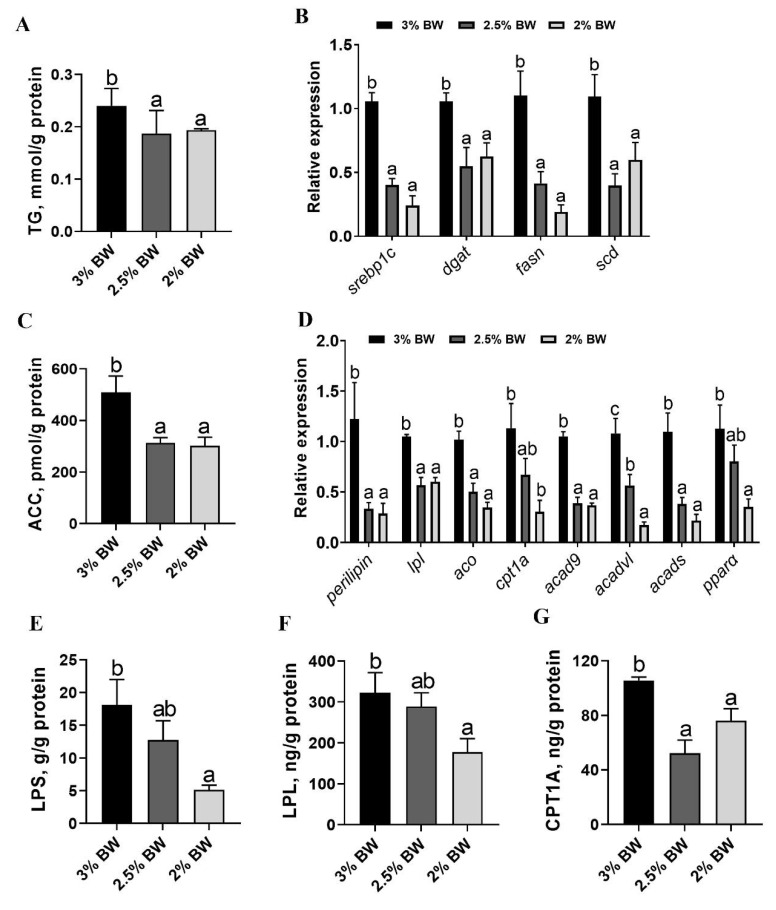
Feed restriction reduced the lipid accumulation and β-oxidation in the liver of channel catfish. (**A**) Triglyceride (TG) content of liver; (**B**) liver transcription levels of lipogenesis-related genes; (**C**) acetyl CoA carboxylase (ACC) content of liver; (**D**) liver transcription levels of lipolysis-related genes; (**E**) lipase (LPS) content of liver; (**F**) lipoprotein lipase (LPL) content of liver; (**G**) carnitine palmitoyltransferase 1A (CPT1A) content of liver. Each data point represents the mean ± SEM of six replicates. Bars assigned different superscripts (a, b and c) are significantly different (*p* < 0.05).

Feed restriction usually reduces lipid deposition by suppressing lipogenesis and promoting lipolysis [25]. Here, we found that the hepatic triglyceride (TG) content significantly decreased in the 2.5% BW and 2% BW groups (*p* < 0.05) (Figure 2A). Accordingly, the transcription levels of key lipogenesis-related genes, *pparα*, *srebp1c*, *dgat*, *fasn* and *scd*, all significantly down-regulated in both the 2.5% BW and 2% BW groups (*p* < 0.05) (Figure 2B). Moreover, the protein concentration of the crucial lipogenesis enzyme, Acetyl CoA carboxylase (ACC), also decreased under feed restriction (*p* < 0.05) (Figure 2C). Unexpectedly, the transcription levels of key lipolysis-related genes, *perilipin*, *lpl*, *aco*, *cpt1a*, *acad9*, *acadvl* and *acads*, almost all down-regulated in both the 2.5% BW and 2% BW groups (*p* < 0.05) (Figure 2D). Furthermore, the protein concentrations of the crucial lipolysis enzymes, Lipase (LPS), Lipoprotein lipase (LPL) and Carnitine palmitoyltransferase 1A (CPT1A), all significantly decreased in the 2% BW group (*p* < 0.05), but only CPT1A significantly decreased in the 2.5% BW group compared to the 3% BW group (Figure 2E–G). These data mean that feed restriction reduces liver lipid accumulation via suppressing lipogenesis and decreasing the fatty acid β-oxidation in the channel catfish under a high water temperature.

### 3.3. Feed Restriction Enhanced Liver Antioxidant Capacity and Improved Thermal Stress State

**Figure 3:**
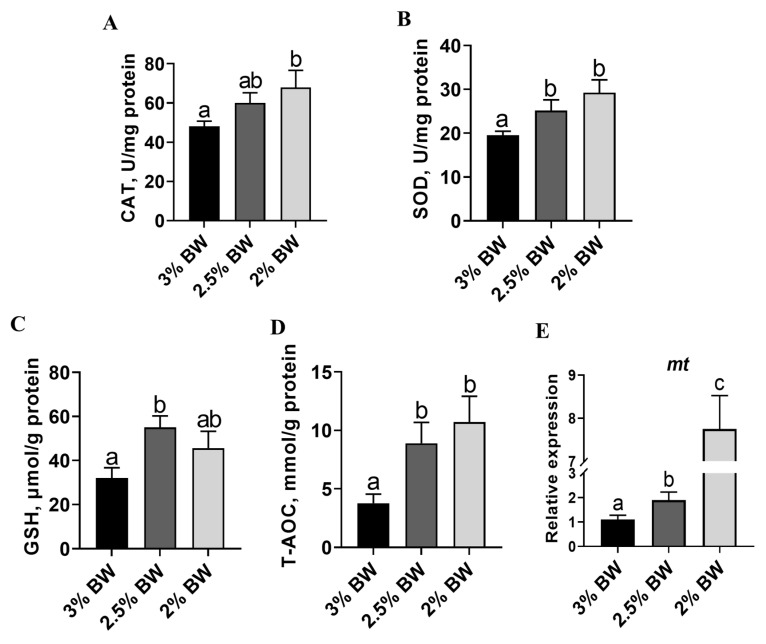
Feed restriction enhanced the liver antioxidant capacity of channel catfish. (**A**) Catalase (CAT) content of liver; (**B**) superoxide dismutase (SOD) content of liver; (**C**) reduced glutathione (GSH) content of liver; (**D**) total antioxidant capacity (T-AOC) of liver; (**E**) liver transcription level of metallothionein (*mt*). Each data point represents the means ± SEM of six replicates. Bars assigned different superscripts (a, b and c) are significantly different (*p* < 0.05).

**Figure 4:**
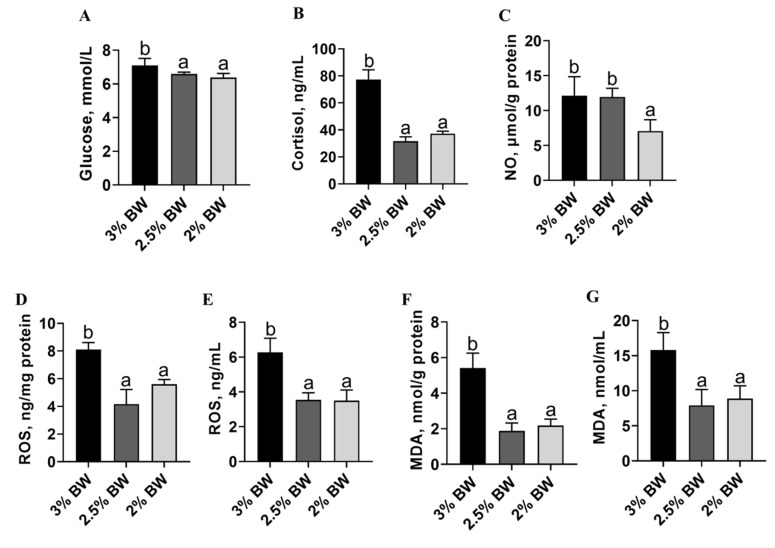
Feed restriction improved thermal stress and peroxidation state in channel catfish. (**A**) Plasma glucose level; (**B**) plasma cortisol level; (**C**) nitric oxide (NO) content of liver; (**D**) reactive oxygen species (ROS) of liver; (**E**) plasma ROS level; (**F**) malondialdehyde (MDA) content of liver; (**G**) MDA content of plasma. Each data point represents the means ± SEM of six replicates. Bars assigned different superscripts (a, b) are significantly different (*p* < 0.05).

Decreased lipid accumulation may contribute to more intensive antioxidant and anti-stress systems in the liver. Hence, we detected the liver antioxidant capacity, stress state and peroxidation degree of the channel catfish under a high water temperature. We found that the activity of the liver-crucial anti-oxidase, catalase (CAT), significantly increased in the 2% BW group (*p* < 0.05) (Figure 3A), while the activity of another crucial anti-oxidase, superoxide dismutase (SOD), significantly increased in both the 2.5% BW and 2% BW groups (*p* < 0.05) (Figure 3B). Moreover, the content of liver-reduced glutathione (GSH) showed a significant increase in the 2.5% BW group (*p* < 0.05), but not in the 2% BW group (Figure 3C). Accordingly, the total antioxidant capacity (T-AOC) of the liver was significantly elevated in both the 2.5% BW and 2% BW groups (*p* < 0.05) (Figure 3D). Interestingly, the transcription level of liver metallothionein (*mt*) also increased in both the 2.5% BW and 2% BW groups (*p* < 0.05) (Figure 3E). We further detected the stress state and peroxidation degree in the livers of channel catfish. We found that the levels of plasma glucose and plasma cortisol both significantly decreased in the 2.5% BW and 2% BW groups (*p* < 0.05) (Figure 4A,B). Nonetheless, the content of liver nitric oxide (NO) only reduced in the 2% BW group (*p* < 0.05) (Figure 4C). Furthermore, the contents of the liver and plasma reactive oxygen species (ROS) and malondialdehyde (MDA) both significantly decreased in the 2.5% BW and 2% BW groups (*p* < 0.05) (Figure 4D–G). These data indicate that feed restriction enhances the liver antioxidant capacity and improves the oxidative stress state of channel catfish under a high water temperature.

### 3.4. Feed Restriction Mitigated the Heat-Induced ER Stress and Apoptosis in Liver

**Figure 5:**
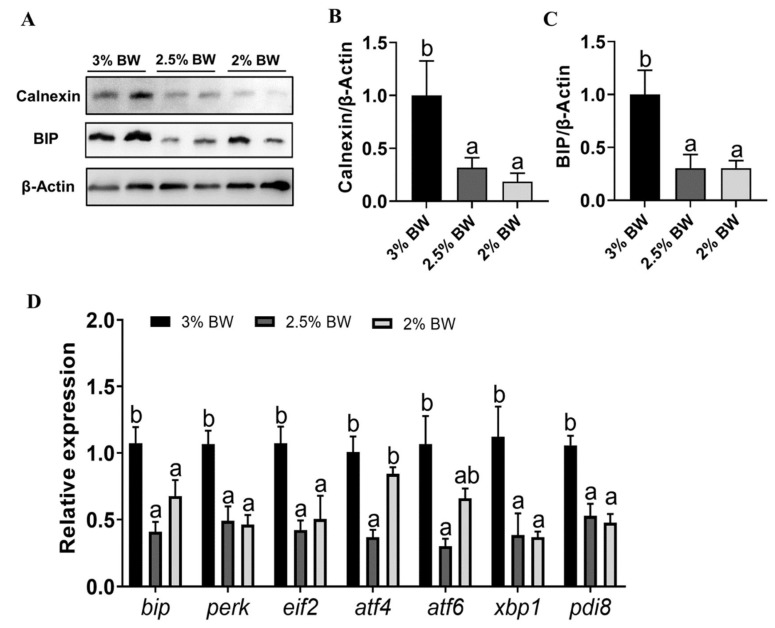
Feed restriction mitigated the chronic thermal stress-induced ER stress in liver of channel catfish. (**A**) Liver Calnexin and BIP protein levels; (**B**) quantification of Calnexin protein level; (**C**) quantification of BIP protein level; (**D**) liver transcription levels of ER stress-related genes. Gels were loaded with 20 mg total protein per lane. Each data point represents the means ± SEM of four replicates (**B**,**C**) or six replicates (**D**). Bars assigned different superscripts (a, b) are significantly different (*p* < 0.05).

**Figure 6:**
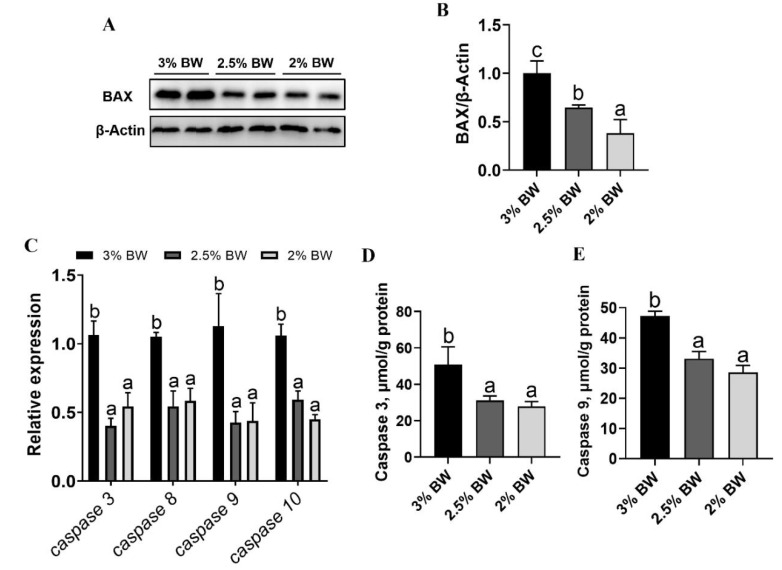
Feed restriction reduced the chronic thermal stress-induced apoptosis in liver of channel catfish. (**A**) Liver BAX protein level; (**B**) quantification of BAX protein level; (**C**) liver transcription levels of apoptosis-related genes; (**D**) Caspase 3 content of liver; (**E**) Caspase 9 content of liver. Each data point represents the means ± SEM of four replicates (**B**) or six replicates (Figure 5C–E). Bars assigned different superscripts (a, b and c) are significantly different (*p* < 0.05).

Since the antioxidant capacity and stress state are closely related to cell ER stress and apoptosis, we further determined the ER stress and apoptosis state in the livers of channel catfish. The results showed that the protein levels of the ER stress makers Calnexin and BIP both significantly reduced in the liver of the 2.5% BW and 2% BW groups (*p* < 0.05) (Figure 5A–C). The transcription levels of ER stress-related genes almost decreased in the 2.5% BW and 2% BW groups (*p* < 0.05), except for activating transcription factor 4 (*atf4*) and activating transcription factor 6 (*atf6*) in the 2% BW group (Figure 5D). For the apoptosis state, we found that the protein levels of BAX significantly reduced in the liver of the 2.5% BW and 2% BW groups (*p* < 0.05) (Figure 6A,B). We further detected the transcription level and activity of the liver Caspase family. The results showed that the transcription levels of *caspase 3*, *caspase 8*, *caspase 9* and *caspase 10* all significantly down-regulated in the 2.5% BW and 2% BW groups (*p* < 0.05) (Figure 6C). Moreover, we found the activities of hepatic Caspase 3 and Caspase 9 also significantly decreased in the 2.5% BW and 2% BW groups (*p* < 0.05) (Figure 6D,E). These data mean that feed restriction mitigates the heat-induced ER stress and apoptosis in the liver of channel catfish under a high water temperature.

### 3.5. Feed Restriction Alleviated Heat-Induced Liver Inflammation and Damages

**Figure 7:**
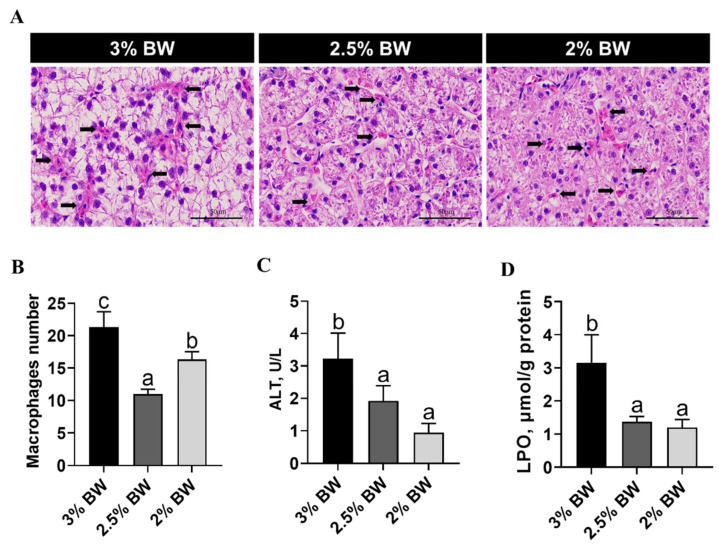
Feed restriction alleviated chronic thermal stress-induced liver inflammation and damages in channel catfish. (**A**) Histological images of liver in channel catfish (H&E stain, arrows indicate macrophages); (**B**) macrophages number; (**C**) plasma alanine aminotransferase (ALT) level; (**D**) lipid peroxidation (LPO) content of liver. Each data point represents the means ± SEM of six replicates. Bars assigned different superscripts (a, b and c) are significantly different (*p* < 0.05).

Given that ER stress and apoptosis ultimately impair liver health, we further evaluated liver inflammation and damages. The results showed that the liver histology of the 2.5% BW and 2% BW groups was better than that in the 3% BW group (Figure 7A), and the number of macrophages significantly decreased in the 2.5% BW and 2% BW groups (*p* < 0.05) (Figure 7A,B). Importantly, the level of plasma alanine aminotransferase (ALT) and the content of hepatic lipid peroxidation (LPO) both significantly reduced in the 2.5% BW and 2% BW groups (*p* < 0.05) (Figure 7C,D). These data indicate that feed restriction alleviates heat-induced liver inflammation and damages in the channel catfish under a high water temperature.

## 4. Discussion

**Figure 8:**
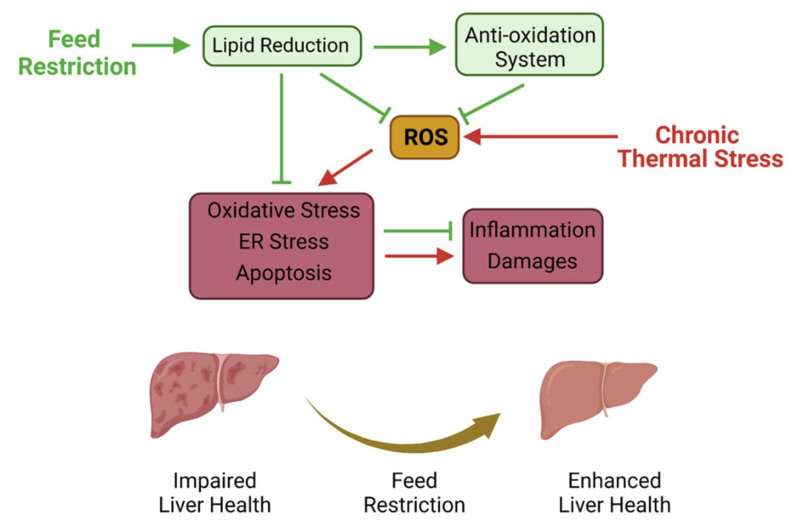
Proposed working model depicting the regulation mechanism of feed restriction alleviating chronic thermal stress-induced liver inflammation and damages in channel catfish.

In China, aquaculture species usually experience a high-temperature period for 2–3 months in summer season. The persistent high temperature deteriorates to a chronic thermal stress, which results in oxidative damages and metabolism disorders in cultured fish [5,6]. Previous studies showed that caloric restriction was an effective approach for reducing oxidative stress [11,34]. Thus, we investigated the effects and potential mechanism of feed restriction on improving thermal stress-induced peroxidation and damages in channel catfish. In the present study, we found that the 2.5% BW feeding ration suppressed the thermal stress response in the liver without causing negative effects on the growth performance of channel catfish under a high water temperature. We further demonstrated that feed restriction enhanced the liver antioxidant capacity and improved the stress state of the channel catfish; that was supposed to be involved in reduced lipid accumulation in liver. Moreover, we found that feed restriction mitigated the heat-induced liver ER stress and apoptosis, which ultimately alleviated liver inflammation and damages of the channel catfish under a high water temperature (Figure 8). Therefore, the present study demonstrated that appropriate feed restriction (2.5% body weight/day) alleviates liver peroxidation and damages via suppressing lipid accumulation in the liver of channel catfish under chronic thermal stress.

Heat shock proteins (HSPs) are a highly conserved family of cellular proteins present in all organisms [35], including fish [36], which are the primary mediators of thermotolerance. HSPs play a role in correcting protein misfolding and defending against the accumulation of immature polypeptides under stress, while protecting cells from protein toxicity and promoting cell growth until conditions improve [37]. Studies on fish also suggested a clear link between thermal stress and heat shock proteins [38]. Here, we found that feed restriction reduced the expression level of HSPs (*hsc70*, *hsp70* and *hsp90*) in the liver of channel catfish under a high water temperature. These results mean the feed restriction can possibly suppress thermal stress in channel catfish. This is similar to a study in blunt snout bream (*Megalobrama amblycephala*), which showed that feed restriction mitigated high carbohydrate-induced oxidative stress [26]. Likewise, the expression of *hsp70* in carp (*Cyprinus carpio*) and rainbow trout (*Oncorhynchus mykiss*) was directly proportional to the transportation pressure [39]. However, feed restriction increased the expression levels of *hsc70* and *hsp70* in the gill tissue of green sturgeon (*Acipenser medirostris*) under heat stress [40]. We suppose that this was due to the excessive feed restriction (12.5% of optimum feeding ration) in that study. In view of the results obtained in this study, the decrease in HSPs may reflect a reduced stress experienced by channel catfish under elevated temperatures. Further investigations should provide more evidence for the relationships between feed restriction and thermal stress in other fish species.

The underlying mechanism for feed restriction to improve thermal stress remains unclear. Lipid accumulation correlated with systemic oxidative stress [41], and obesity was thought to be a state of chronic oxidative stress [42]. Moreover, HSPs not only function as a housekeeper and cell protector, but also as a companion for lipid metabolism [36,37]. Therefore, we speculate that improved thermal stress is involved in the decreased lipid deposition, since the feed restriction reduced the liver lipid accumulation and the CF value of channel catfish, which were consistent with a study in grass crap (*Ctenopharyngodon idella*) [25]. A previous study also illustrated that decreased lipid deposition alleviated heat stress in the liver of broilers [43]. Inversely, heat stress was proven to promote lipid accumulation by inhibiting the AMPK-PGC-1α pathway in preadipocytes [44]. Thus, an appropriate feed restriction is beneficial for maintaining the total lipid homeostasis in the thermal stress state. Furthermore, increased β-oxidation induces mitochondrial dysfunction and elevates the generation of ROS and MDA [45]. Here, we found that feed restriction enhanced the liver antioxidant capacity (CAT, SOD, GSH and T-AOC) and improved the oxidative stress state (glucose, cortisol, ROS and MDA) of the channel catfish. A previous study showed that the non-alcoholic fatty liver disease (NAFLD) was involved in the decreased antioxidant capacity and increased oxidation stress induced by accumulation of lipids in the liver [46,47]. In fish, a high-fat diet induced an over-deposition of lipids in liver, which also impaired the antioxidant capacity and caused ROS damages [48,49]. Importantly, feed restriction or caloric restriction ameliorated the oxidative stress via reducing lipid accumulation in the liver [34,50]; the same is true in fish [26,51]. However, a study showed that feed restriction (lower than 75% satiation) decreased antioxidant capacity and triggered oxidative stress in sobaity (*Sparidentex hasta*) and yellowfin seabream (*Acanthopagrus latus*) [52]. We speculate that this is due to the excessively low feeding level, and the feeding experiment was not in a thermal condition in that study. Hence, we think feed restriction improved the stress state and enhanced the antioxidant capacity through a reduction in the lipid accumulation in the liver of channel catfish under a high water temperature.

It is known that thermal stress and peroxidation induce ER stress and apoptosis [53,54,55], which ultimately cause tissue damages. Various forms of cellular stress induce misfolding events and then lead to the aggregation of proteins within endoplasmic reticulum, causing ER stress [56]. Here, we found the biomarkers of ER stress (Calnexin, BIP, *perk* and *atf6*) all downregulated in the feed restriction groups. These data indicate that feed restriction can reduce thermal stress-induced ER stress, which is consistent with a study in rodents [50]. Apoptosis is a form of programmed cell death regulated by the Caspases family, and the activated Caspases can induce apoptosis to remove damaged cells in tissues [57]. Here, we found that the members of the Caspases family and BAX protein were suppressed by feed restriction, which indicated that feed restriction can alleviate thermal stress-induced cell apoptosis in the liver of channel catfish. However, a study demonstrated that short-term caloric restriction increased liver apoptosis, which was not associated Caspase levels [58]. We attribute to that study that it is not a thermal stress-induced liver apoptosis; there may exist a different regulation mechanism. Moreover, ER stress and apoptosis are closely related to tissue inflammation and damages [59,60]. Notably, the thermal stress-induced liver inflammation and damages were well improved by the feed restriction in the present study. Given these results, we propose that feed restriction alleviates heat-induced liver inflammation and damages via mitigating ER stress and apoptosis in the channel catfish under a high water temperature.

## 5. Conclusions

Taken together, the present study demonstrated that an appropriate feed restriction alleviated liver peroxidation and damages via suppressing lipid accumulation in the liver of cultured fish under chronic thermal stress (Figure 8). Given the growth performance, antioxidant capacity and stress state, 2.5% body weight/day was recommended to improve the liver health of channel catfish in the summer season. These findings first highlight the feasibility of feed restriction on enhancing the antioxidant and anti-stress capacities of fish suffering from thermal stress and lay the foundation for research into health care improved by feeding strategies in aquaculture.

## Figures and Tables

**Table 1:** Primers used for gene expressions assay by real-time PCR.

Target Genes	Forward (5′-3′)	Reverse (5′-3′)	Accession Numbers
*aco*	AGACCTGAACTTTCTGTCCCG	GCTGGACACCATAGGGATGAA	XM_017482134.1
*acad9*	CTGAAAAGAGTGGCCGATACA	CGTCATTGTTCTCTGGGGAAT	XM_017450491.1
*acadvl*	CTGTGCGATTGACTTGTATGC	CTCTACCACAGCTGCAGAAAT	XM_017472293.1
*acads*	TCACTCAGCTTGCAGGATTAC	ATGACTGCTTAGCTGGGAAAG	XM_017489904.1
*atf4*	GATTCTGATGGCTGACACCTT	GGTCAAAATCACTGAGGTCGA	XM_017482287.1
*atf6*	AGGAGTTTGAGGTGATGATGC	TCTACTCCAATTGCTGACACG	XM_017455892.1
*bip*	GAGACAGGCCAAGATTGAGAG	TTCTGAGTTGGAGCCTGATTG	XM_017460312.1
*cpt1a*	ACCACATCCCAATCTGCCTG	CCTGAAGTGAGCAAGCTGGA	XM_017485733.1
*caspase3*	GTGTGTGTGATCCTAAGCCAT	CAAATCTGTTCCACGACAAGC	NM_001201081.1
*caspase8*	TATGAGGAAGAGGACACGGAA	CGCAATGAGGAAATCTGCATC	XM_017480625.1
*caspase9*	TGCTTCCTCTGAAACCAACAA	GATCTGAATCGTCTCTCCAGC	XM_017487604.1
*caspase10*	ACGTGGAGTTCTTCTGTGATG	AGCTGTTCGAGGTTCTTACAC	XM_017470757.1
*dgat*	AAGAAATTCCCCGGGATCAAG	ACAGGAGGTAATCGATGGAGT	NM_001201076.1
*eif2*	CATTCTTGGAGGTTCAGAGGG	CATTCAAACGTGCCATCCAAA	XM_017468034.1
*fasn*	CTGGTCAGAGCAACTACGGTT	CTACATCACCGATAGCACCCC	XM_017483746.1
*hsc70*	CAAGATCAGTGACGAGGACAAG	GGTTACAGACTTTCTCCAGTTCC	XM_017489684.1
*hsp70*	CTTGATGTTACCCCTCTGTCTCT	TCAGAGTAGGTGGTGAAAGTCTG	NM_001200273.1
*hsp90*	ATCTGAAGGAGGATCAGACAGAG	CGCTCCTTCTCTACAAAGAGTGT	NM_001329313.1
*lpl*	CTGGACGGTTACTGGCATGT	GGCAGCTGAGGTTGGGTAAT	XM_017478439.1
*mt*	CTGCAAATGCTCAAACTGCCA	AGCACTTGGAATCGCAGGTAT	NM_001200077.1
*perilipin*	GGACATTGACCATTGCTCGC	ACCTCGCGTGTTTATGAGCA	XM_017472797.1
*pparα*	TCCGCAAGCCTTTCAGTGAT	AGCAAGGTGTCATCAGGGTG	XM_017494485.1
*perk*	GCAGCAAAACATTCGTGTGTA	TGGTACGATTCGGTCTCTGTA	XM_017455330.1
*pdi8*	GAATGAACCGAGGCGATATGA	CAAACCACATTTCCCAGCATC	XM_017482284.1
*scd*	GTTTGGACGTGTGGAAATGAC	TGTGTATGTTGAAGCTGAGGG	XM_017464581.1
*srebp1c*	ACTGGCTGCAAGGAGATGAC	TGTCTCTGAACAGGCTGCTG	XM_017452596.1
*xbp1*	TCTGGTCAGGAAAGAGAAGGT	AGCTCTGGTTCAATGATGTCC	XM_017452343.1
*β-actin*	GGATCTGTATGCCAACACTGT	CAGGTGGGGCAATGATCTTAA	XM_017454668.1

Note: aco, acyl-CoA oxidase 1; acad9, acyl-CoA dehydrogenase family member 9; acadvl, acyl-CoA dehydrogenase, very long chain; acads, acyl-CoA dehydrogenase, C-2 to C-3 short chain; atf4, activating transcription factor 4; atf6, activating transcription factor 6; bip, 78 kDa glucose-regulated protein; caspase3, caspase 3; caspase8, caspase 8; caspase9, caspase 9; caspase10, caspase 10; cpt1a, carnitine palmitoyltransferase 1A; dgat, diacylglycerol O-acyltransferase 2; eif2, GCN1 activator of EIF2AK4; fasn, fatty acid synthase; hsc70, heat shock cognate 71 kDa protein; hsp70, heat shock protein 70; hsp90, heat shock protein 90; lpl, lipoprotein lipase; mt, metallothionein; pdi8, protein disulfide isomerase family A member 8; perilipin, perilipin; perk, estrogen receptor 1; pparα, peroxisome proliferator activated receptor alpha; scd, stearoyl-CoA de-saturase; srebp1c, sterol regulatory element binding transcription factor 1; xbp1, X-box binding protein 1; β-actin, actin beta.

**Table 2:** Effects of feed restriction on the growth performance of channel catfish (*Ictalurus punctatus*).

	3% BW	2.5% BW	2% BW	*p*-Value
IBW, g	35.50 ± 0.06	35.50 ± 0.06	35.50 ± 0.05	*p* > 0.05
FBW, g	99.86 ± 1.04 ^b^	97.56 ± 1.55 ^b^	83.84 ± 0.67 ^a^	*p* < 0.05
WG, %	181.28 ± 2.83 ^b^	174.81 ± 4.24 ^b^	136.33 ± 1.82 ^a^	*p* < 0.05
SGR, %/d	1.15 ± 0.01 ^b^	1.11 ± 0.01 ^b^	0.99 ± 0.03 ^a^	*p* < 0.05
FE, %	73.82 ± 1.29 ^a^	84.91 ± 1.59 ^b^	87.93 ± 0.78 ^b^	*p* < 0.05
FR, %BW/d	3.02 ± 0.05 ^c^	2.48 ± 0.03 ^b^	2.04 ± 0.02 ^a^	*p* < 0.05
SR, %	98.96 ± 0.50	99.25 ± 0.50	98.75 ± 0.68	*p* > 0.05

Note: Data indicate the mean values of six replicates per treatment. Mean values with different superscripts in a row are significantly different (one-way ANOVA, *p* < 0.05). IBW, initial body weight; FBW, final body weight; WG, weight gain rate; SGR, specific growth rate; FE, feed efficiency; FR, feeding rate; SR, survival rate.

**Table 3:** Effects of feed restriction on the physical index of channel catfish (*Ictalurus punctatus*).

	3% BW	2.5% BW	2% BW	*p*-Value
HSI, %	1.47 ± 0.05 ^b^	1.14 ± 0.10 ^a^	1.17 ± 0.05 ^a^	*p* < 0.05
VSI, %	8.43 ± 0.41 ^b^	8.01 ± 0.17 ^ab^	7.45 ± 0.20 ^a^	*p* < 0.05
CF, g/cm^3^	1.40 ± 0.05 ^b^	1.26 ± 0.04 ^a^	1.32 ± 0.04 ^ab^	*p* < 0.05

Note: Data indicate the mean values of six replicates per treatment. Mean values with different superscripts in a row are significantly different (one-way ANOVA, *p* < 0.05). HSI, hepatosomatic index; VSI, viscerosomatic index; CF, condition factor.

## Data Availability

All data generated or analyzed during this study are included in this article.
